# Cost burden and net monetary benefit loss of neonatal hypoglycaemia

**DOI:** 10.1186/s12913-021-06098-9

**Published:** 2021-02-05

**Authors:** Matthew J. Glasgow, Richard Edlin, Jane E. Harding

**Affiliations:** 1 Liggins Institute, University of Auckland, Private Bag 92019, Grafton, Auckland, 1142 New Zealand; 2 School of Population Health, University of Auckland, Auckland, New Zealand

**Keywords:** Cerebral palsy, Epilepsy, Learning disorder, Newborn infant, Vision disorder

## Abstract

**Background:**

Neonatal hypoglycaemia is a common but treatable metabolic disorder that affects newborn infants and which, if not identified and treated adequately, may result in neurological sequelae that persist for the lifetime of the patient. The long-term financial and quality-of-life burden of neonatal hypoglycaemia has not been previously examined.

**Methods:**

We assessed the postnatal hospital and long-term costs associated with neonatal hypoglycaemia over 80 year and 18 year time horizons, using a health-system perspective and assessing impact on quality of life using quality-adjusted life year (QALYs). A decision analytic model was used to represent key outcomes in the presence and absence of neonatal hypoglycaemia.

**Results:**

The chance of developing one of the outcomes of neonatal hypoglycaemia in our model (cerebral palsy, learning disabilities, seizures, vision disorders) was 24.03% in subjects who experienced neonatal hypoglycaemia and 3.56% in those who do did not.

Over an 80 year time horizon a subject who experienced neonatal hypoglycaemia had a combined hospital and post-discharge cost of NZ$72,000 due to the outcomes modelled, which is NZ$66,000 greater than a subject without neonatal hypoglycaemia. The net monetary benefit lost due to neonatal hypoglycaemia, using a value per QALY of NZ$43,000, is NZ$180,000 over an 80 year time horizon.

**Conclusions:**

Even under the most conservative of estimates, neonatal hypoglycaemia contributes a significant financial burden to the health system both during childhood and over a lifetime. The combination of direct costs and loss of quality of life due to neonatal hypoglycaemia means that this condition warrants further research to focus on prevention and effective treatment.

**Supplementary Information:**

The online version contains supplementary material available at 10.1186/s12913-021-06098-9.

## Background

Neonatal hypoglycaemia is a common but treatable metabolic disorder that affects newborn infants, most often in the first 24 h after birth. It is typically asymptomatic, and if not identified and treated adequately, may result in neurological sequelae that persist for the lifetime of the patient [1]. The overall incidence is estimated to be up to 15% of all infants, and 50% in infants with risk factors such as being born small, large, preterm, or to a mother with diabetes [2, 3].

Although severe symptomatic neonatal hypoglycaemia has been recognised since 1937 [4], controversy and knowledge gaps in understanding this condition persist, particularly pertaining to its definition, the degree and duration of hypoglycaemia that may result in complications [3], and the risk of complications associated with asymptomatic disease [5]. There is also great variation in definitions of outcomes, tools for assessing the presence and severity of outcomes, the age at which assessments are made, and the characteristics of populations in which the outcomes are measured.

Short-term costs have been described previously for infants at increased risk of neonatal hypoglycaemia [6], but there remains a paucity of high quality prospective evidence examining the post-discharge outcomes of neonatal hypoglycaemia [5, 7], and their costs. We have undertaken an economic analysis to compare the costs and utilities for subjects who experienced neonatal hypoglycaemia and those who did not, with the objective of quantifying the total cost burden due to neonatal hypoglycaemia, and, via net monetary benefit loss estimations, an indication of the impact of longer term outcomes useful for future economic evaluations of preventative treatments.

## Methods

We assessed the postnatal hospital and long-term costs associated with neonatal hypoglycaemia over 80 year and 18 year time horizons, and assessing impact on quality of life using quality-adjusted life year (QALYs), from the perspective of the New Zealand healthcare system, where health and disability services, including inpatient and outpatient public hospital and primary care services are funded or subsidised by the government [8]. A decision analytic model was used to represent key outcomes in the presence and absence of neonatal hypoglycaemia.

### Classification of outcomes

In order to determine the outcomes of neonatal hypoglycaemia and their respective probabilities (prevalences) we searched Medline, EMBASE, and CINAHL databases combining: 1) the diagnosis of neonatal hypoglycaemia with; 2) previously reported neurodevelopmental or neurological outcomes of neonatal hypoglycaemia or the standardised assessment tools used to identify them; or 3) Subject Heading Terms for outcome measures, quality of life measures, outcome assessments, or health status indicators (Additional file 1). Publications cited within the identified studies were also reviewed.

Our initial literature search, including hand searching, yielded 2530 reports, of which 2446 were excluded on title and abstract searching, and the remaining 84 studies were used to identify outcomes related to neonatal hypoglycaemia, including candidate clinical outcomes for inclusion in our model (Table 1). Of these, 43 studies reported the probabilities of at least one outcome, or a probability could be readily calculated from a relative risk or absolute number. Thirty-five studies were excluded, 22 because they reported populations with significant confounders or comorbidities, small populations, or did not include sufficient information to calculate prevalence in the hypoglycaemic subgroup. A further 13 studies were excluded because of a high risk of bias. This was independently assessed by two authors, using the Joanna Briggs Institute Checklist for Prevalence Studies [22], and converted to a numeric risk-of-bias score based on the ratio of checklist responses indicating high risk of bias to those indicating low risk of bias, excluding those not applicable to the publication being considered. The remaining 8 publications with a score < 50% were considered at low risk of bias and included in our analysis (Additional file 2). Some publications contributed more than one prevalence value per outcome due to, for instance, different cohorts or different outcome subsets.

**Table 1:** Candidate outcomes and reported issues

Developmental Domain	Reported Issue (with sources)
Cognitive	Low IQ [9]
	Cognitive dysfunction [10]
	Impaired perceptive performance [11]
	Cognitive delay [12]
Language	Verbal skills delay [9]
	Speech language delay [12]
Motor	Impaired coordination/motricity [11]
	Cerebral palsy [12]
Social-Emotional	Hyperactivity and inattention [13]
Adaptive Behaviour	Impairment of adaptability and motivation [14]
Executive Function	Impairment of recognition memory [15]
	Working memory deficits [16, 17]
	Impairment of explicit memory (recall) after a delay [18]
Growth	Lower body weight [9]
	Suboptimal head growth [9, 12]
Visual	Occipital lobe injury (MRI) [19, 20]
	Blindness or impaired visual acuity [12]
	Other specific visual impairment, including squint, visual field defect, cortical visual impairment, immature visual attention and tracking, visuo-spatial difficulties [12]
Hearing	Deafness or impaired hearing
Neurological	White matter abnormalities [12]
	Seizures/epilepsy [12, 21]

If no published reports describing prevalence were found in our search, that outcome was not included in our model. This resulted in a final list of five key outcomes with prevalence data able to be included in our model:

- Cerebral palsy [1, 23, 24]
- Learning disabilities (mild-moderate learning disorders, language development disorders, intellectual disability) [1, 23–28]
- Severe learning disabilities (severe or global developmental delay) [23]
- Epilepsy (seizures beyond those during the initial episodes of hypoglycaemia) [23, 29]
- Vision disorders (including blindness and central processing disorders) [23]

For intellectual and/or learning disabilities, we categorised mild-to-moderate intellectual disability as IQ of 70–85, or a description of functional level implying an IQ in that range (e.g., possibly requiring educational support during school age, but able to live independently and perform activities of daily living without ongoing support). We categorised severe intellectual disability as an IQ < 70, described as having severe or profound learning or intellectual disabilities, or requiring full or part time homecare support for supervision, assistance with self-care or communication. Two studies were excluded as they report outcome prevalences at 2 years of age in cohorts that overlap with that reported by McKinlay et al. [23] at 4.5 years of age (McKinlay et al. 2015 [30], Harris et al. [31]). Data from the older age were selected in order to capture morbidities, such as some learning disabilities, which are less reliably assessed at a younger age.

The weighted mean prevalence for each outcome was calculated as the sum of all qualifying cases across all included studies divided by the sum of the total population across all included studies. This varied from 2 Cases within a population of 270 for vision disorders through to 7604 Cases within a population of 1,421,813 for epilepsy (Table 2). The size of the combined population, and overall number of cases, informed the parameters used to represent the beta distribution of these prevalences in our stochastic analysis.

**Table 2:** Prevalences of morbidities due to neonatal hypoglycaemia

Single health-state outcomes:	Number of prevalence reports^a^	Sum of cases	Sum of study populations	Weighted mean prevalence	Minimum prevalence	Maximum prevalence
Cerebral palsy	4 [1, 23, 24]	53	1020	5.20%	0.38%	14.89%
Childhood epilepsies and convulsions	3 [23, 29]	7604	1,421,813	0.53%	0.52%	4.58%
Learning disabilities	7 [1, 23–28]	205	1314	15.60%	2.38%	54.00%
Severe learning disabilities	1 [23]	9	278	3.24%	3.24%	3.24%
Vision disorders/blindness	1 [23]	2	270	0.74%	0.74%	0.74%

^a^Some publications contributed more than one prevalence value per outcome

Since individuals can have more than one outcome of interest, we examined the original data from two studies that have reported the outcomes included in our model in children with increased risk for hypoglycaemia (the Children With Hypoglycaemia and Their Later Development [CHYLD] Study [23] and the Protein, Insulin, and Neonatal Outcomes [PIANO] Study [28]. In the CHYLD + PIANO cohorts, the prevalence of any multiple-issue health state (i.e., two or more concurrent morbidities) was 2.59%. Not all combinations of outcomes occurred in these cohorts. The combinations of cerebral palsy with learning disorders (any severity), and blindness/vision disorders with learning disorders (any severity) each occurred with a higher frequency than expected by chance (Fisher’s Exact Test 2-sided *p* values of 0.001 and 0.004 respectively). Within our analysis, however, estimates of mean prevalence for different outcomes are treated as independent due to data limitations (including the low expected counts for most outcomes).

Prevalences of the outcomes in the general population, independent of neonatal hypoglycaemia status, were sought using similar strategies for outcomes (Additional file 1) and costs (Additional file 3). Large meta-analyses were selected to determine the overall prevalences for cerebral palsy [32], epilepsy [33], intellectual disability [34], and vision impairment [35].

### Costs

We searched Medline, EMBASE, and CINAHL for published direct medical costs associated with the selected outcomes, regardless of aetiology. We considered studies for inclusion if they reported a standard deviation or confidence interval for costs, and provided transparent estimates of included cost components and sample size. We made the assumption that costs for an outcome were independent of the aetiology of that outcome.

For post-discharge costs, reports from Australia or New Zealand populations were prioritised, with other geographical populations included in the absence of Australasian data. For patients with cerebral palsy and learning disabilities, we used estimates that included total health expenditure (inpatient costs, outpatient costs, medication costs) from the United States [36]. Australian costs were used for patients with learning disabilities [37], epilepsy [38], and visual impairment [39, 40] (Table 3).

**Table 3:** Post-discharge health expenditures per patient for hypoglycaemia-related outcomes

Outcome	Outcome subgroup	Population	Cost components	Cost (per patient, mean annual, 2018 NZ$)
Cerebral Palsy	All	Medicaid-enrolled patients < 17 years of age, United States	Total health care expenditure (inpatient costs, outpatient costs, medications), US$ (2005 data)	$41,332 [36]
	Cerebral palsy, no intellectual disability			$31,211 [36]
Severe intellectual disorders/ learning disabilities	IQ 50–69	Families with children with intellectual disability, Australia	Annual government assistance; out-of-pocket health and home care expenses, AU$ (2012 data)	$15,532 [37]
	IQ 35–49			$25,317 [37]
	IQ < 35			$17,857 [37]
Epilepsy	Epilepsy	All patients, Australia	Direct health care costs (hospital costs, medication costs, other), US$ (1990 data)	$5196 [38]
Vision disorders	Impaired visual acuity, mixed aetiologies	All patients, Australia	Total health-related cost (informal care and support; medicines, products, and equipment; health and community services; and other expenses), US$ (2009 data)	$3124 [39]
	Mixed aetiologies	All patients, Australia	Direct health system costs, based on health service utilisation, AU$ (2000–2004 data)	$5377 [40]

To convert NZ$ to US$ multiply by 0.6938

Annualised costs were converted from published currencies to NZ$ and US$ using purchasing power parities (PPP) [41] and then corrected for inflation to 2018 levels (end of second quarter) using the Personal Consumption Expenditures (PCE) health-by-function index [42], which includes out-of-pocket health expenditure and personal consumption of health services paid on behalf by third party payers [43] (Table 3). Costs as used as input parameters with their respective distributions and distribution parameters are shown in Additional file 4.

The costs calculated by Doran et al. [37] were all considered to relate to severe intellectual disability. Kancherla et al. [36] estimated costs separately for cerebral palsy with and without intellectual disability. However, their distinctions between levels of severity of intellectual disability and their exclusion of learning disorders mean their definitions of mild, moderate, and severe cases are not well aligned with those in our model, and thus we have used their cost estimates for cerebral palsy without intellectual disability only.

Because our definition of mild-to-moderate intellectual disability/learning disorders describes subjects who may need additional educational support but who are unlikely to incur medical costs beyond those of the general population, we did not attribute any direct health-related costs to this group.

The definition of vision disorder was visual acuity < 6/12 of any aetiology [39, 40]. The populations considered for assessing cost of visual disorders included all ages, including patients with age-related visual problems.

The overall lifetime cost was considered to be the sum of the initial postnatal hospital costs, and the cumulative annual total post-discharge healthcare expenditure specifically for each outcome over the time horizons of the analysis, discounted at 3.5% [44, 45] for costs incurred in timeframes greater than 1 year. Postnatal hospital costs were based on the lengths-of-stay in a general postnatal ward and a neonatal intensive care unit (NICU), and their costs, as described previously [6], and were converted and inflated to 2018 NZ$ using the methods outlined above. The average cost of a postnatal hospital stay used for an infant with neonatal hypoglycaemia was NZ$7500, and for an infant without neonatal hypoglycaemia was NZ$1100.

### Utility weights

For the base analysis, we used the catalogue of Kwon et al. [46] (Table 4), and for sensitivity analyses we used the published paediatric condition utility weight catalogues of Petrou and Kupek [47] and Carroll and Downs [48] (Additional file 5). Utility weights were discounted at the same rate and in the same manner as for costs.

**Table 4:** Utility weights from Kwon et al. [43]

Event Tree Model Outcomes	Category Descriptions (ICD-10 groups)	Utility Weight (95% CI)
Learning disabilities	Cognitive impairment (F06)	0.48 (0.45–0.50)
Severe learning disabilities/Global developmental delay	Mental retardation (F72)	0.28 (0.21–0.34)
Cerebral palsy	Cerebral palsy (G80)	0.35 (0.28–0.42)
Childhood epilepsies and convulsions	Epilepsy (G40)	0.55 (0.31–0.79)
Vision disorders/blindness	Visual disturbances and blindness (H54)	0.55 (0.48–0.62)

Few of the outcomes reported are mutually exclusive, and disabilities can occur together. For the modelled scenarios involving comorbid outcomes, the utility of the most severe component (i.e., the outcome with the lowest utility) was used to determine impact on quality of life. Because the utility rank order of the outcomes differed between the different utility weight sets used in our sensitivity analyses, this meant that the outcome selected to represent the utility of the most severe component of comorbid outcomes sometimes differed between the base case and sensitivity analyses. For utility weight sets that provided more than one utility weight value for a specified outcome (e.g., with separations based on severity), a mean value was used.

### Analysis

The analysis considered all 24 possible combinations of outcomes i.e. as cerebral palsy (yes/no), epilepsy (yes/no), vision disorders (yes/no) and learning disabilities (severe, mild-moderate, and none) (Table 5).

**Table 5:** Input parameters for base analysis

Cerebral palsy	Epilepsy	Visual disorders	Learning disability		Probability		Utility [46]	Post-discharge cost^a^
			Severe	Mild-moderate	Hypoglycaemia	No hypoglycaemia		
–	–	–	–	–	75.9656%	96.4347%	0.876	$ 0.00
–	–	–	–	Yes	14.6016%	0.9601%	0.476	$31,784.45
–	–	–	Yes	–	3.0326%	0.0606%	0.276	$31,784.45
–	–	Yes	–	–	0.5663%	1.6802%	0.547	$4250.65
–	–	Yes	–	Yes	0.1089%	0.0167%	0.476	$31,784.45
–	–	Yes	Yes	–	0.0226%	0.0011%	0.276	$31,784.45
–	Yes	–	–	–	0.4048%	0.6192%	0.552	$5196.11
–	Yes	–	–	Yes	0.0778%	0.0062%	0.476	$31,784.45
–	Yes	–	Yes	–	0.0162%	0.0004%	0.276	$31,784.45
–	Yes	Yes	–	–	0.0030%	0.0108%	0.547	$5196.11
–	Yes	Yes	–	Yes	0.0006%	0.0001%	0.476	$31,784.45
–	Yes	Yes	Yes	–	0.0001%	0.0000%	0.276	$24,748.93
Yes	–	–	–	–	4.1669%	0.2029%	0.348	$31,211.41
Yes	–	–	–	Yes	0.8009%	0.0020%	0.348	$80,894.69
Yes	–	–	Yes	–	0.1663%	0.0001%	0.276	$80,894.69
Yes	–	Yes	–	–	0.0311%	0.0035%	0.348	$31,211.41
Yes	–	Yes	–	Yes	0.0060%	0.0000%	0.348	$80,894.69
Yes	–	Yes	Yes	–	0.0012%	0.0000%	0.276	$31,211.41
Yes	Yes	–	–	–	0.0222%	0.0013%	0.348	$80,894.69
Yes	Yes	–	–	Yes	0.0043%	0.0000%	0.348	$80,894.69
Yes	Yes	–	Yes	–	0.0009%	0.0000%	0.276	$80,894.69
Yes	Yes	Yes	–	–	0.0002%	0.0000%	0.348	$31,211.41
Yes	Yes	Yes	–	Yes	0.0000%	0.0000%	0.348	$80,894.69
Yes	Yes	Yes	Yes	–	0.0000%	0.0000%	0.276	$80,894.69

^a^Per patient, mean annual, 2018 NZ dollars (to convert NZ$ to US$ multiply by 0.6938)

The costs and utility/QALYs associated with hypoglycaemia were calculated as a weighted sum of all these possibilities. We calculated net monetary benefits (NMB) for the subjects with and without neonatal hypoglycaemia, and the net monetary benefit loss due to hypoglycaemia as the difference between these two values, using values per quality-adjusted life year (λ) of NZ$43,000 and NZ$14,000.

We conducted a stochastic analysis using 100,000 runs drawing from the estimated distributions of input parameters (beta distributions for prevalence and utility values, lognormal distribution for costs). Credible intervals were calculated for the cost differences and net monetary benefit lost due to neonatal hypoglycaemia, as the 2.5 and 97.5% percentiles for those parameters across the 100,000 runs of the stochastic analysis using the PERCENTILE.EXC function of Microsoft Excel. For input parameters where the standard deviation was not reported in, or able to be calculated from, the source material, their relationship between the expected value and standard deviation was presumed to be comparable to the other input parameters of the same type.

We conducted the following one-way sensitivity analyses:

- substituting the alternative catalogues of utilities [47, 48] for childhood diseases
- substituting a multiplicative method to estimate the utility values in multiple health state outcomes
- discount rates of 0 and 5%
- calculating the costs of multiple health state outcomes using the sum of the costs of all included outcomes
- using only the lowest published prevalence for each major outcome
- using prevalences for vision disorder and epilepsy after neonatal hypoglycaemia that are equivalent to the respective prevalences in the population without neonatal hypoglycaemia.

We estimated the financial implications of neonatal hypoglycaemia, in terms of the healthcare costs difference and the net monetary benefit lost due to neonatal hypoglycaemia, for the New Zealand population, and extrapolated to the United States population, in their respective 2018 currencies (to convert NZ$ to US$ multiply by by 0.6938). These estimates were based on an incidence of neonatal hypoglycaemia (< 2.6 mmol/L) of 15.3% (30% of all infants born at increased risk and 51% of these experiencing neonatal hypoglycaemia [2]).

## Results

### Base analysis

In our base analysis, the chance of developing one of the outcomes in our model was 24.03% in subjects who had experienced neonatal hypoglycaemia and 3.56% in those who had not (Additional file 6).

**Fig. 1:**
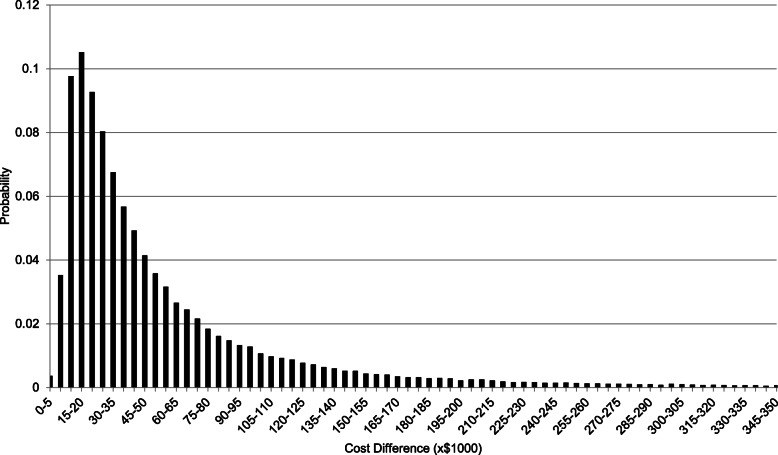
Distribution of cost difference between hypoglycaemia and controls (80 year time horizon)

Over an 80 year time horizon a subject who had experienced neonatal hypoglycaemia had a combined discounted hospital and post-discharge cost of NZ$72,000, which is NZ$66,000 greater than a subject without neonatal hypoglycaemia (Table 6). However, there is significant uncertainty in this cost difference, with the 95% credible interval estimated in our stochastic analysis spanning NZ$8800–300,000 (Fig. 1).

**Table 6:** Costs, cost difference, and net monetary benefit for different input parameters, 80 year time horizon

Utility Source and Method	Cost method	Discount Rate	Prevalence Source	Cost, NZ$, Hospital and Post-Discharge			Utility, QALYs		Net Monetary Benefit Loss, NZ$	
				Neonatal Hypoglycaemia	No Neonatal Hypoglycaemia	Difference	Neonatal Hypoglycaemia	No Neonatal Hypoglycaemia	λ = $43 k	λ = $14 k
Kwon et al., minimum value	Outcome with greatest cost	3.5%	Mean, all outcomes	$72,233.42	$6175.89	$66,057.53	21.25	23.89	$180,252.41	$103,775.53
Kwon et al., minimum value	Sum of all outcomes	3.5%	Mean, all outcomes	$71,847.80	$6137.82	$65,709.97	21.25	23.89	$179,906.51	$104,723.87
Kwon et al., product of values	Outcome with greatest cost	3.5%	Mean, all outcomes	$71,928.23	$6155.35	$65,772.88	21.19	23.89	$182,624.71	$111,480.44
Petrou et al., minimum value	Outcome with greatest cost	3.5%	Mean, all outcomes	$72,478.70	$6193.92	$66,284.79	22.08	25.22	$201,874.00	$99,124.63
Carrol et al., TTO, minimum value	Outcome with greatest cost	3.5%	Mean, all outcomes	$72,147.79	$6166.69	$65,981.10	25.09	27.39	$165,411.90	$98,896.91
Carrol et al., SG, minimum value	Outcome with greatest cost	3.5%	Mean, all outcomes	$71,866.26	$6188.04	$65,678.22	25.09	27.39	$165,335.51	$287,808.34
Kwon et al., minimum value	Outcome with greatest cost	0.0%	Mean, all outcomes	$193,484.20	$15,707.46	$177,776.74	61.41	69.04	$507,875.19	$78,883.76
Kwon et al., minimum value	Outcome with greatest cost	5.0%	Mean, all outcomes	$55,431.26	$4859.70	$50,571.56	15.79	17.76	$135,510.35	$33,429.72
Kwon et al., minimum value	Outcome with greatest cost	3.5%	Minimum, all outcomes	$30,945.31	$6208.07	$24,737.24	23.29	23.89	$50,818.36	$106,689.74
Kwon et al., minimum value	Outcome with greatest cost	3.5%	Mean, with vision disorders and epilepsy values equal to population without hypoglycaemia	$73,723.28	$6188.28	$67,534.99	21.18	23.89	$184,998.44	$103,775.53

*Abbreviations*: *QALY* quality-adjusted life year, *λ* willingness-to-pay, *SG* standard gamble, *TTO* time trade-off

To convert NZ$ to US$ multiply by 0.6938

Over the first 18 years of life, the cost difference between a subject with and without neonatal hypoglycaemia is NZ$36,000, (Table 7) and spans a 95% credible interval of NZ$7600-150,000.

**Table 7:** Costs, cost difference, and net monetary benefit for different input parameters, 18 year time horizon

Utility Source and Method	Cost method	Discount Rate	Prevalence Source	Cost, NZ$, Hospital and Post-Discharge			Utility, QALYs		Net Monetary Benefit Loss, NZ$	
				Neonatal Hypoglycaemia	No Neonatal Hypoglycaemia	Difference	Neonatal Hypoglycaemia	No Neonatal Hypoglycaemia	λ = $43 k	λ = $14 k
Kwon et al., minimum value	Outcome with greatest cost	3.5%	Mean, all outcomes	$39,431.82	$3590.91	$35,840.91	10.48	11.78	$92,149.00	$54,610.28
Kwon et al., minimum value	Sum of all outcomes	3.5%	Mean, all outcomes	$39,241.22	$3572.01	$35,669.20	10.48	11.78	$91,978.93	$54,438.77
Kwon et al., product of values	Outcome with greatest cost	3.5%	Mean, all outcomes	$39,281.48	$3581.04	$35,700.45	10.45	11.78	$93,319.29	$54,905.74
Petrou et al., minimum value	Outcome with greatest cost	3.5%	Mean, all outcomes	$39,552.32	$3599.79	$35,952.54	10.89	12.43	$102,809.90	$58,237.90
Carrol et al., TTO, minimum value	Outcome with greatest cost	3.5%	Mean, all outcomes	$39,389.59	$3586.38	$35,803.21	12.38	13.51	$84,831.79	$52,145.75
Carrol et al., SG, minimum value	Outcome with greatest cost	3.5%	Mean, all outcomes	$39,250.22	$3596.91	$35,653.32	12.37	13.51	$84,792.87	$52,033.33
Kwon et al., minimum value	Outcome with greatest cost	0.0%	Mean, all outcomes	$49,365.56	$4368.33	$44,997.22	13.82	15.53	$119,268.94	$69,753.46
Kwon et al., minimum value	Outcome with greatest cost	5.0%	Mean, all outcomes	$36,101.08	$3333.21	$32,767.87	9.42	10.59	$83,435.22	$49,656.72
Kwon et al., minimum value	Outcome with greatest cost	3.5%	Minimum, all outcomes	$19,072.44	$3606.86	$15,465.57	11.48	11.78	$28,324.82	$19,752.29
Kwon et al., minimum value	Outcome with greatest cost	3.5%	Mean, with vision disorders and epilepsy values equal to population without hypoglycaemia	$40,166.02	$3597.08	$36,568.94	10.44	11.78	$94,489.58	$55,875.70

*Abbreviations*: *QALY* quality-adjusted life year, *λ* willingness-to-pay, *SG* standard gamble, *TTO* time trade-off

To convert NZ$ to US$ multiply by 0.6938

**Fig. 2:**
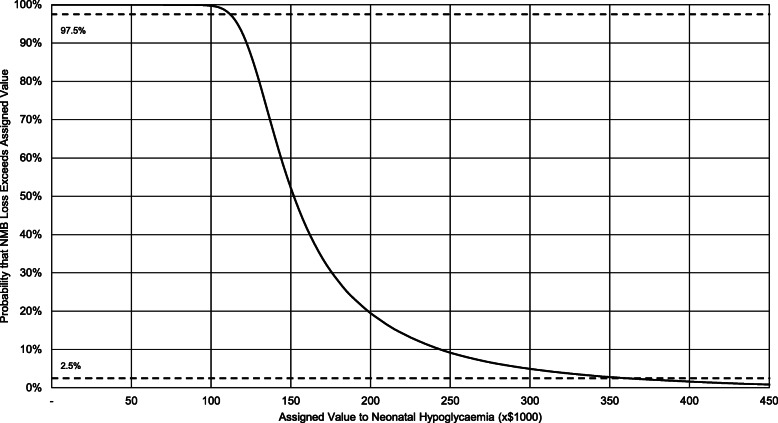
Decumulative probability of NMB distribution, displaying probability that NMB loss exceeds an assigned value per case of neonatal hypoglycaemia

In addition to these cost differences, neonatal hypoglycaemia also leads to health losses. If the health lost due to neonatal hypoglycaemia is valued at NZ$43,000 per QALY and added to the cost impacts, then the expected net monetary loss due to neonatal hypoglycaemia is around NZ$190,000 over an 80 year time horizon. In New Zealand, the national pharmaceutical agency (PHARMAC) does not specify a cost threshold per QALY for determining if an intervention is cost-effective [44]. If the health lost due to neonatal hypoglycaemia is valued at the higher level of NZ$72,000 per QALY, the expected net monetary loss due to neonatal hypoglycaemia is around NZ$260,000. This is the value to avoiding an additional case of neonatal hypoglycaemia and can guide the evaluation of treatments addressing these risks. Figure 2 illustrates the uncertainty around this figure, and shows a 95% credible interval of NZ$110,000-420,000.

### Sensitivity analyses

The mean net monetary benefit loss attributable to neonatal hypoglycaemia was not greatly affected by using different catalogues of utility values for childhood diseases, or using the approach of multiplying relevant utility values (Tables 6 and 7).

One-way sensitivity analyses that employed 0 and 5% discount rates for costs and utilities altered the mean net monetary benefit loss due to neonatal hypoglycaemia to NZ$510,000 and NZ$140,000 respectively. The conservative approach of using only the lowest outcome prevalences reduced the mean loss to NZ$51,000 over an 80 year time horizon with a λ of NZ$43,000, but even over the 18 year time horizon with a λ of NZ$14,000, the net monetary loss of neonatal hypoglycaemia persisted, at NZ$20,000.

### National costs of neonatal hypoglycaemia

In New Zealand, where the study of dextrose gel prophylaxis was undertaken [49], there are approximately 58,000 live births per year [50]. This equates to an estimated 8874 cases of neonatal hypoglycaemia per year, with an associated cost of NZ$590,000,000. Thus, a prophylactic strategy that achieved a 21% reduction in cases of neonatal hypoglycaemia would result in an 80-year cost saving of NZ$120,000,000, or a net monetary benefit saving of NZ$320,000,000 over an 80 year time horizon.

In the United States there are approximately 3,855,500 live births [51] and an estimated 589,892 cases of neonatal hypoglycaemia per year, costing US$27,000,000,000 annually. In a study of hypoglycaemia prevention with dextrose gel, the relative risk of hypoglycaemia was 0.79 compared with placebo [49]. Although we note the differences in the structure of the health systems between the two countries, in the United States, a 21% reduction in cases would therefore result in an 80-year cost saving of approximately US$5,400,000,000, or a net monetary benefit saving of US$15,000,000,000 over an 80 year time horizon.

## Discussion

Neonatal hypoglycaemia is a common condition that affects up to 15% of all newborns. Both the healthcare-related costs of, and impact on quality of life due to, the long-term outcomes of neonatal hypoglycaemia accrue over the lifetime of the subject. A paucity of data pertaining to the post-discharge outcomes of neonatal hypoglycaemia [5] has meant that quantification of these burdens is difficult, and there have been calls for well-designed studies to examine the association between neonatal hypoglycaemia and long-term neurodevelopmental outcomes [5, 52]. Importantly, the economic impact of the long-term outcomes of neonatal hypoglycaemia also have not previously been investigated. We have used currently available data to estimate the cost difference between subjects with and without neonatal hypoglycaemia, and the net monetary benefit lost, which includes an estimate of the impact on quality of life attributable to neonatal hypoglycaemia.

We estimated that the cost difference between an infant who develops neonatal hypoglycaemia and one who does not is NZ$66,000 over an 80 year time horizon, with NZ$36,000 of this attributable within the first 18 years. The net monetary benefit lost due to neonatal hypoglycaemia, which reflects the level the healthcare system would be willing to pay to prevent cases, using a willingness-to-pay value of NZ$43,000 per quality-adjusted life year, is NZ$180,000 per patient over an 80 year time horizon, and NZ$92,000 per patient over an 18 year time horizon. The bulk of this cost is accrued after discharge from the initial post-natal hospital stay for both time horizon cost calculations. In New Zealand, and by extrapolation in the United States, these accumulate to significant national costs and net monetary benefit losses due to neonatal hypoglycaemia over the lifetime of the patient. Prevention of this condition is difficult, but early feeding is recommended, and buccal dextrose gel prophylaxis looks promising [49]. Our data suggest that a prophylactic strategy that achieved a reduction of even a modest proportion of cases would result in substantial cost savings and quality of life improvements in the population.

To the best of our knowledge, this is the first economic analysis of the long-term outcomes of neonatal hypoglycaemia. Strengths of our study include the use of standard decision analysis modelling methodologies, and systematic literature reviews to determine the input parameters of our model. In particular, a systematic approach was used to select studies reporting the prevalences of outcomes of neonatal hypoglycaemia.

Further, in order to reflect the uncertainty and broad distributions of input parameters in our model, we have performed our analysis using a conservative approach. The sensitivity analyses used variations in the source and methods associated with the input parameters, including very conservative analyses using minimum values for the outcome prevalences, and an upper range for discount rate of 5% per annum. In addition, we have focused only on the direct costs to the healthcare system. The inclusion of other societal costs, particularly those borne by the education system or in the form of other government-funded support, and families of affected individuals, and indirect costs, would increase the overall financial costs of this condition.

Our model incorporates a number of simplifications to overcome data limitations, particularly pertaining to the prevalence of outcomes. The outcomes we incorporated into our model are limited to those for which prevalence data were available, and for which impact on quality of life can be represented by utility weights available in the selected paediatric utility weight catalogues. The prevalence values we selected for inclusion span a fairly wide distribution for each outcome, despite selection on the basis of low risk of bias. This is predominantly due to the small study populations and numbers of cases, with the exception of data pertaining to epilepsy [29]. The exclusion of outcomes such as decreased body weight, suboptimal head growth during infancy, and radiological findings such as white matter abnormalities observed by MRI scanning, will result in conservative cost and utility estimations. Ongoing long-term clinical studies investigating the relationship between the severity and frequency of neonatal hypoglycaemia and subsequent neurodevelopmental outcomes [23] will contribute to more accurate estimations of the prevalence of such complications, and data that can ultimately be incorporated into future iterations of economic analyses of neonatal hypoglycaemia.

Specific challenges were encountered in estimating cost parameters for our model, including that it was necessary to combine sources across countries, despite acknowledged differences in approaches to healthcare funding and payment. Notably challenging were the limitations in estimating costs of learning disabilities in children, including carer benefits/opportunity lost, the costs borne by the education system, and the affected individual’s capacity to earn income thereafter. Our model therefore excludes indirect costs, and costs outside of the healthcare system. In many instances, particularly for mild or moderate learning disabilities, there may be negligible or very little additional cost to the healthcare system (and for this reason, the costs for these were set to zero in the model), with the majority of financial impact coming in the form of supplementary or specialised teaching support financed by the education sector or privately by family or caregivers. The implication of this approach is that, by focusing on healthcare system costs, our model considerably underestimates the overall societal costs.

Further complicating the estimation of the costs of all severities of learning disabilities, as for many other conditions of childhood, is the fact that direct costs are predominantly encountered earlier in life, although indirect costs and opportunity costs may manifest during adulthood. In our model, we have employed flat cost input parameters across the lifespan, but have presented results for both 18 year (childhood) and 80 year (lifetime) time horizons. In our base analysis, and in all sensitivity analyses that used a discount rate of 3.5%, the 80 year time horizon cost difference and the net monetary benefit loss is approximately double that of the 18 year time horizon. Thus, the application of discounting means that subjects in our model encounter more of their overall healthcare costs earlier. This also reflects the reality that a larger proportion of overall healthcare costs for childhood conditions may occur early, although we note that the extent to which later costs for pharmaceutical therapy, and ongoing outpatient follow-up and hospital treatment, span a wide range.

Although we used existing catalogues of utility values for childhood conditions, it is worth noting that quality of life indices are more challenging to determine accurately in the paediatric population than in adult populations. Reasons for this include, but are not limited to, the frequent requirement to use a proxy respondent (parent or caregiver) to determine impact [53, 54], rapid developmental changes affecting the relevance of health status indicators across age ranges and developmental states [54], and a lack of validated multi-attribute utility instruments for the very young (< 5 years of age) [53].

We modelled the outcomes present in comorbid states as being independent. Our calculated proportions of comorbidities approximates those of other reports of the prevalence of comorbid childhood chronic conditions, where estimates have been made that fewer than 5% of children younger than 18 years have two or more chronic conditions, and fewer than 1% have three or more chronic conditions [55]. The ratio of comorbid outcomes to single-health-state outcomes is thus relatively small, reducing the impact of uncertainties in estimation of probabilities and costs, and in the uncertainties introduced by the use of a multiplicative approach to calculating the combined prevalence.

Similarly, as the number of comorbidities increases, cumulative deteriorations in health status measures will be observed. Although a number of approaches have been proposed for estimation of the utility of joint health states [56–58], there is no gold standard for their derivation from single health-state utilities [57]. When modelling the utility of comorbidities in our event tree, in the absence of utility data for specific combinations of chronic conditions [57], particularly those manifesting during childhood, we used the utility of the most severe component (i.e., a “minimum estimation” approach), rather than applying a multiplicative model, as the former has been demonstrated to provide a more accurate estimation [59]. We included the latter method as a sensitivity analysis in order to assess the impact of more conservative multiple health state outcome utility values, and found little impact on the overall cost differences or net monetary benefit loss due to neonatal hypoglycaemia.

Learning disabilities and developmental delay, in particular, as comorbid health states, generally increase in prevalence as the number of other chronic conditions increases [55], and can be proportional in severity to the accumulated health-burden-over-time of the accompanying other chronic childhood conditions [60]. Although the utilisation of health services increases under these circumstances, these subjects are often represented within the distributions of costs, particularly when estimations have been made by analysing third-party payment systems [61]. This is in part due to the fact that, in the United States, the costs associated with intellectual disability are not necessarily coded in Medicaid claims unless this has a direct impact on the primary diagnosis [36]. Kancherla et al. [36] sought to resolve costs in a more granular manner by separating out their cost estimates of cerebral palsy with and without intellectual disability, but noted that under-diagnosis of intellectual disability may mean that children with severe intellectual disability are overrepresented in the cohorts, resulting in an overestimation of the cost of intellectual disability that co-occurs with chronic conditions such as cerebral palsy [36].

Although some of the clinical outcomes of neonatal hypoglycaemia may have an impact on lifespan, we have not explicitly modelled this. Patients with intellectual disability form the largest group of individuals with negative clinical outcomes due to neonatal hypoglycaemia within our model. No difference in mortality was observed in a large, 35-year population-based cohort study of persons with intellectual disability [62]. The impact of any premature mortality due to other neonatal hypoglycaemia-related outcomes, such as epilepsy [63], which is more likely to be evident over the 80 year time horizon than the 18 year time horizon, is mitigated by discounting, wherein long-term costs are borne early, with late costs being devalued cumulatively.

We have sought to mitigate these limitations and challenges by incorporating the wide distributions of the cost, prevalence, and utility input parameters into stochastic versions of our model, and by undertaking sensitivity analyses that were intentionally conservative.

## Conclusions

The long-term financial and quality-of-life burden of neonatal hypoglycaemia has not been previously examined. We have analysed the impact of the long-term outcomes of neonatal hypoglycaemia using a decision analytic model.

Even under the most conservative of conditions, our estimation of the cost of neonatal hypoglycaemia both over childhood and over a lifetime shows that neonatal hypoglycaemia contributes a significant financial burden to the health system. The combination of direct costs and loss of quality of life due to neonatal hypoglycaemia means that this condition warrants further research to focus on prevention and effective treatment.

## Supplementary Information

**Additional file 1: Supplementary Table 1.** Search strategy for neonatal hypoglycaemia outcomes (Medline and Embase).

**Additional file 2: Supplementary Figure 1.** PRISMA flow diagram - prevalences of neonatal hypoglycaemia outcomes.

**Additional file 3: Supplementary Table 2.** Search strategy for costs (Medline and Embase).

**Additional file 4: Supplementary Table 3.** Costs of single health state conditions.

**Additional file 5: Supplementary Table 4.** Utility weights.

**Additional file 6: Supplementary Table 5.** Prevalences of single health state conditions.

## Data Availability

All data generated or analysed during this study are included in this published article [and its supplementary information files], and/or are available from the corresponding author on reasonable request.

## Abbreviations

CHYLD: Children With Hypoglycaemia and Their Later Development Study
IQ: Intelligence quotient
MRI: Magnetic resonance imaging
NICU: Neonatal intensive care unit
NMB: Net monetary benefits
PCE: Personal consumption expenditures
PIANO: Protein, Insulin, and Neonatal Outcomes Study
PPP: Purchasing power parities
QALY: Quality-adjusted life years
SG: Standard gamble
TTO: Time trade off
