# Supplementary material for: Cost burden and net monetary benefit loss of neonatal hypoglycaemia
Source: BMC Health Serv Res. 2021 Feb 5;21:121. doi: 10.1186/s12913-021-06098-9 (PMC7863541; doi:10.1186/s12913-021-06098-9)
Supplement: Supplementary file 1 — Additional file 1: Supplementary Table 1. Search strategy for neonatal hypoglycaemia outcomes (Medline and Embase). [file 12913_2021_6098_MOESM1_ESM.pdf]

Supplementary Table 1: Search strategy for neonatal hypoglycaemia outcomes (Medline and Embase)

1	(infant, newborn/ OR infant, low birth weight/ OR infant, premature/ OR (infant* OR newborn* OR neonat*).mp) AND (hypoglycemia/ or hypoglyc*.mp)
2	behavioral symptoms/ OR child behavior/ OR communication/ OR language/ OR literacy/ OR child development/ OR language development/ OR mental competency/ OR memory disorders/ OR intellectual disability/ OR perceptual disorders/ OR neurocognitive disorders/ OR cognition disorders/ OR neurodevelopmental disorders/ OR epilepsy/ OR hearing disorders/ OR vision disorders/ OR cerebral palsy/ OR motor disorders/ OR muscle hypertonia/ OR muscle hypotonia/ OR muscle weakness/ OR myotonia/ OR spasm/ OR paralysis/ OR (cognitive or delay OR IQ OR mental development OR Stanford-Binet OR McCarthy OR language OR speech OR verbal OR motor OR palsy OR motricity OR hyperactivity OR inattention OR executive function* OR memory OR growth OR visual OR occipital OR seizure* OR epilepsy).mp
3	"quality of life" OR "outcome assessment (health care)" OR health status/ OR health status indicators/ OR (health outcome* or outcome measure*).mp
4	1 AND 2 (2365 results)
5	(1 AND 2) OR (1 AND 3) (2525 results)

Note: searches in other databases followed a similar approach, differing in syntax alone
