# Supplementary figures and images for: Cost burden and net monetary benefit loss of neonatal hypoglycaemia
Source: BMC Health Serv Res. 2021 Feb 5;21:121. doi: 10.1186/s12913-021-06098-9 (PMC7863541; doi:10.1186/s12913-021-06098-9)

**Supplementary Figure 1: PRISMA Flow Diagram - Prevalences of Neonatal Hypoglycaemia Outcomes**

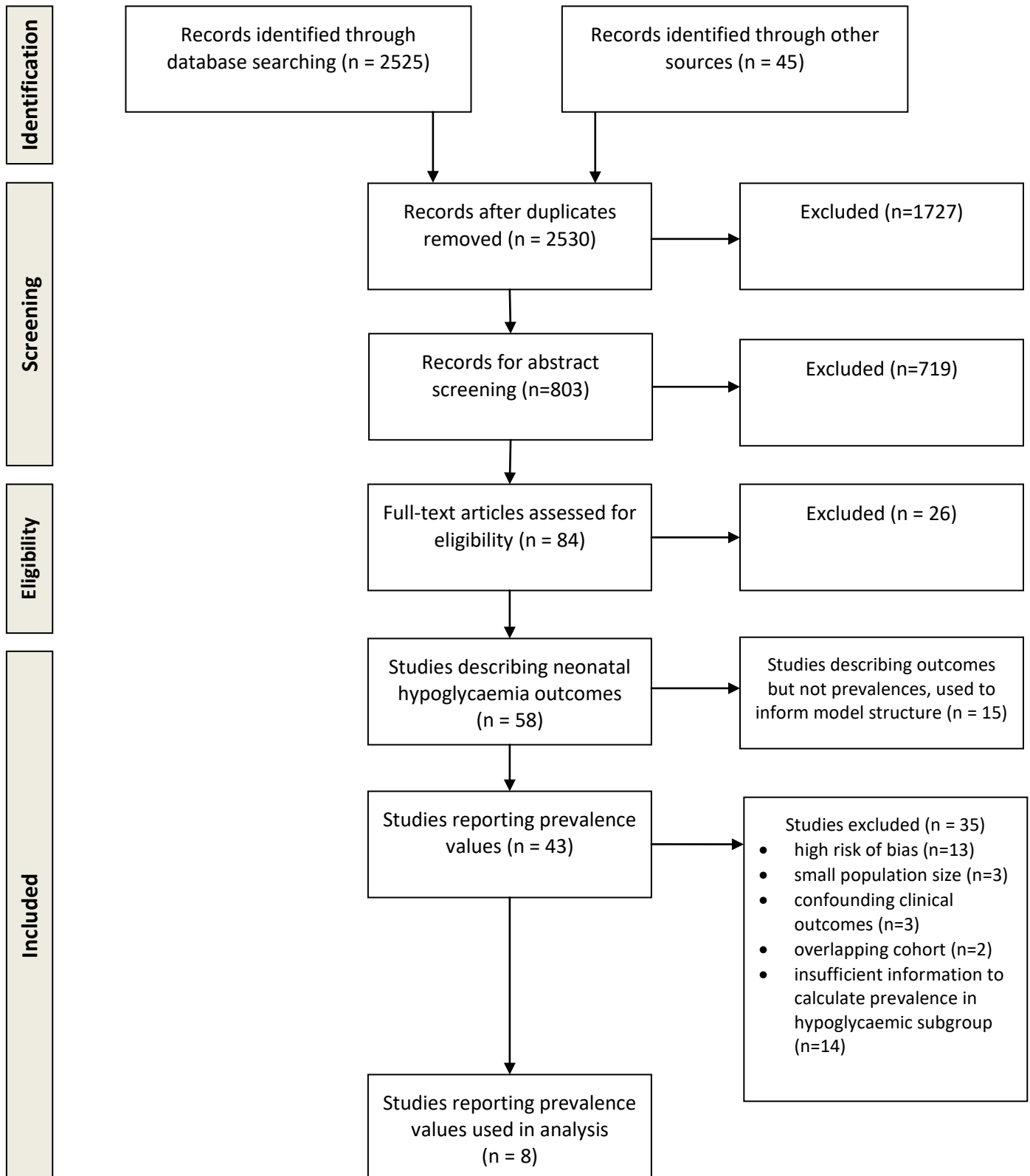

Supplement: Supplementary file 2 — Additional file 2: Supplementary Figure 1. PRISMA flow diagram - prevalences of neonatal hypoglycaemia outcomes. [file 12913_2021_6098_MOESM2_ESM.pdf]
