# Supplementary material for: Cost burden and net monetary benefit loss of neonatal hypoglycaemia
Source: BMC Health Serv Res. 2021 Feb 5;21:121. doi: 10.1186/s12913-021-06098-9 (PMC7863541; doi:10.1186/s12913-021-06098-9)
Supplement: Supplementary file 3 — Additional file 3: Supplementary Table 2. Search strategy for costs (Medline and Embase). [file 12913_2021_6098_MOESM3_ESM.pdf]

Supplementary Table 2: Search strategy for costs (Medline and Embase)

Cerebral palsy	cerebral palsy/ or cerebral palsy.mp
Learning disabilities	learning disorders/ or language development disorders/ or intellectual disability/ or (learn* or intellect*).mp.
Epilepsy	exp epilepsy/ or (epilep* or seizure*).mp
Vision disorders	vision disorders/ or blind*.mp
Economics filter	economics/ OR "costs and cost analysis"/ OR cost allocation/ OR cost-benefit analysis/ OR cost control/ OR cost savings/ OR cost of illness/ OR cost sharing/ OR "deductibles and coinsurance"/ OR medical savings accounts/ OR health care costs/ OR direct service costs/ OR drug costs/ OR employer health costs/ OR hospital costs/ OR health expenditures/ OR capital expenditures/ OR value of life/ OR exp economics, hospital/ OR exp economics, medical/ OR economics, nursing/ OR economics, pharmaceutical/ OR exp "fees and charges"/ OR exp budgets/ OR (low adj cost).mp OR (high adj cost).mp OR (health?care adj cost$).mp OR (fiscal or funding or financial or finance).tw OR (cost adj estimate$).mp OR (cost adj variable).mp OR (unit adj cost$).mp OR (economic$ or pharmacoeconomic$ or price$ or pricing).tw
Cost of illness filter	exp "Cost of Illness"/
Regionalisation	exp australia/ or exp new zealand/
