# Supplementary material for: Cost burden and net monetary benefit loss of neonatal hypoglycaemia
Source: BMC Health Serv Res. 2021 Feb 5;21:121. doi: 10.1186/s12913-021-06098-9 (PMC7863541; doi:10.1186/s12913-021-06098-9)
Supplement: Supplementary file 4 — Additional file 4: Supplementary Table 3. Costs of single health state conditions. [file 12913_2021_6098_MOESM4_ESM.pdf]

Supplementary Table 3: Costs of single health state conditions

	Cost (NZ$)	Distribution	μ	σ
Postnatal hospital stay, neonatal hypoglycaemia	$7,523.31	Derived, from lognormal distributions of raw data [1]		
Postnatal hospital stay, no neonatal hypoglycaemia	$1,075.17	Derived, from lognormal distributions of raw data [1]		
Cerebral palsy, per annum [2]	$31,211.41	Lognormal	9.04	1.37
Epilepsy, per annum [3]	$5,196.11	Lognormal	7.25	1.37
Severe learning disorder, per annum [4]	$20,736.66	Lognormal	8.63	1.37
Vision disorders, per annum [5,6]	$4,250.24	Lognormal	7.05	1.37
Note: NZ$ to US$ conversion factor = 0.6938				

1. Glasgow MJ, Harding JE, Edlin R. Cost analysis of treating neonatal hypoglycemia with dextrose gel. J Pediatr 2018.
2. Kancherla V, Amendah DD, Grosse SD, Yeargin-Allsopp M, Van Naarden Braun K. Medical expenditures attributable to cerebral palsy and intellectual disability among Medicaid-enrolled children. Res Dev Disabil 2012;33:832-40.
3. Kotsopoulos IA, Evers SM, Ament AJ, de Krom MC. Estimating the costs of epilepsy: an international comparison of epilepsy cost studies. Epilepsia 2001;42:634-40.
4. Doran CM, Einfeld SL, Madden RH, et al. How much does intellectual disability really cost? First estimates for Australia. J Intellect Dev Disabil 2012;37:42-9.
5. Keeffe JE, Chou S-L, Lamoureux EL. The cost of care for people with impaired vision in Australia. Arch Ophthalmol-Chic 2009;127:1377-81.
6. Taylor HR, Pezzullo ML, Keeffe JE. The economic impact and cost of visual impairment in Australia. Br J Ophthalmol 2006;90:272-5.
