# Supplementary material for: Cost burden and net monetary benefit loss of neonatal hypoglycaemia
Source: BMC Health Serv Res. 2021 Feb 5;21:121. doi: 10.1186/s12913-021-06098-9 (PMC7863541; doi:10.1186/s12913-021-06098-9)
Supplement: Supplementary file 5 — Additional file 5: Supplementary Table 4. Utility weights. [file 12913_2021_6098_MOESM5_ESM.pdf]

**Supplementary Table 4: Utility weights**

| Condition | Utility | Distribution | α | β |
|---|---|---|---|---|
| Utility decrement, based on Kwon et al[1] | | | | |
| Cerebral Palsy | 0.528 | Beta | 99.75 | 89.17 |
| Childhood epilepsy and convulsions | 0.324 | Beta | 4.37 | 9.12 |
| Learning disabilities, mild and moderate | 0.400 | Beta | 469.98 | 704.97 |
| Learning disabilities, severe | 0.600 | Beta | 134.45 | 89.63 |
| Vision disorders/blindness | 0.329 | Beta | 55.02 | 112.21 |
| Utility decrement, based on Petrou et al[2] | | | | |
| Cerebral Palsy | 0.652 | Beta | 36.91 | 19.70 |
| Childhood epilepsy and convulsions | 0.602 | Beta | 32.18 | 21.28 |
| Learning disabilities, mild and moderate | 0.510 | Beta | 32.93 | 31.64 |
| Learning disabilities, severe | 0.549 | Beta | 31.83 | 26.14 |
| Vision disorders/blindness | 0.452 | Beta | 17.68 | 21.44 |
| Based on Carrol and Downs, Time Trade Off approach[3] | | | | |
| Mild mental retardation | 0.83 | Beta | 1.38 | 0.28 |
| Moderate mental retardation | 0.79 | Beta | 1.69 | 0.45 |
| Severe mental retardation | 0.51 | Beta | 0.73 | 0.71 |
| Mild cerebral palsy | 0.88 | Beta | 1.69 | 0.23 |
| Moderate cerebral palsy | 0.76 | Beta | 1.29 | 0.41 |

| | | | | |
|---|---|---|---|---|
| Severe cerebral palsy | 0.55 | Beta | 0.70 | 0.57 |
| Mild seizure disorder | 0.86 | Beta | 1.49 | 0.24 |
| Moderate seizure disorder | 0.88 | Beta | 1.04 | 0.14 |
| Severe seizure disorder | 0.71 | Beta | 1.30 | 0.53 |
| Mild bilateral vision loss | 0.91 | Beta | 1.15 | 0.11 |
| Moderate bilateral vision loss | 0.86 | Beta | 1.49 | 0.24 |
| Based on Carrol and Downs, Standard Gamble approach[3] | | | | |
| Mild mental retardation | 0.84 | Beta | 1.38 | 0.28 |
| Moderate mental retardation | 0.79 | Beta | 1.69 | 0.45 |
| Severe mental retardation | 0.59 | Beta | 0.73 | 0.71 |
| Mild cerebral palsy | 0.87 | Beta | 1.69 | 0.23 |
| Moderate cerebral palsy | 0.76 | Beta | 1.29 | 0.41 |
| Severe cerebral palsy | 0.6 | Beta | 0.70 | 0.57 |
| Mild seizure disorder | 0.85 | Beta | 1.49 | 0.24 |
| Moderate seizure disorder | 0.84 | Beta | 1.04 | 0.14 |
| Severe seizure disorder | 0.7 | Beta | 1.30 | 0.53 |
| Mild bilateral vision loss | 0.89 | Beta | 1.15 | 0.11 |
| Moderate bilateral vision loss | 0.85 | Beta | 1.49 | 0.24 |

1. Kwon J, Kim SW, Ungar WJ, Tsiplova K, Madan J, Petrou S. A systematic review and meta-analysis of childhood health utilities. Med Decis Making 2017:272989X17732990.
2. Petrou S, Kupek E. Estimating preference-based Health Utilities Index Mark 3 utility scores for childhood conditions in England and Scotland. Med Decis Making 2009;29:291-303.
3. Carroll AE, Downs SM. Comprehensive cost-utility analysis of newborn screening strategies. Pediatrics 2006;117:S287-95.
