# Supplementary material for: Cost burden and net monetary benefit loss of neonatal hypoglycaemia
Source: BMC Health Serv Res. 2021 Feb 5;21:121. doi: 10.1186/s12913-021-06098-9 (PMC7863541; doi:10.1186/s12913-021-06098-9)
Supplement: Supplementary file 6 — Additional file 6: Supplementary Table 5. Prevalences of single health state conditions. [file 12913_2021_6098_MOESM6_ESM.pdf]

Supplementary Table 5: Prevalences of single health state conditions

Condition	Prevalence, mean (deterministic)	Distribution	α	β
With neonatal hypoglycaemia				
Cerebral Palsy[1-3]	5.20%	Beta	52.94	965.06
Childhood epilepsies and convulsions[1,4]	0.53%	Beta	7535.60	1414275.40
Learning disabilities, mild and moderate[1-3,5-8]	15.60%	Beta	204.67	1107.33
Learning disabilities, severe[1]	3.24%	Beta	8.94	267.06
Vision disorders/blindness[1]	0.74%	Beta	1.98	266.02
Without neonatal hypoglycaemia				
Cerebral Palsy[9]	0.21%	Beta	751.34	357030.89
Childhood epilepsies and convulsions[10]	0.64%	Beta	183.56	28586.88
Learning disabilities, all[11]	1.04%	Beta	623.09	59462.53
Learning disabilities, mild and moderate	0.97%	Derived from Learning disabilities, all		
Learning disabilities, severe	0.06%	Derived from Learning disabilities, all		
Vision disorders/blindness, < 50 years of age	1.71%	Derived from Vision disorders/blindness subpopulations		
Blindness, < 50 years of age, male[12]	0.08%	Beta	2.14	2676.14

Vision disorders, moderate-severe, < 50 years of age, male[12]	0.74%	Beta	2.94	394.85
Vision disorders, mild, < 50 years of age, male[12]	0.81%	Beta	1.62	198.50
Blindness, < 50 years of age, female[12]	0.09%	Beta	2.08	2304.43
Vision disorders, moderate-severe, < 50 years of age, female[12]	0.82%	Beta	2.84	343.64
Vision disorders, mild, < 50 years of age, female[12]	0.89%	Beta	1.58	176.27

1. McKinlay CJD, Alsweiler JM, Anstice NS, et al. Association of neonatal glycemia with neurodevelopmental outcomes at 4.5 years. JAMA Pediatr 2017;171:972-83.
2. Lucas A, Morley R, Cole TJ. Adverse neurodevelopmental outcome of moderate neonatal hypoglycaemia. BMJ 1988;297:1304-8.
3. Tin W, Brunskill G, Kelly T, Fritz S. 15-year follow-up of recurrent "hypoglycemia" in preterm infants. Pediatrics 2012;130:e1497-503.
4. Razaz N, Tedroff K, Villamor E, Cnattingius S. Maternal body mass index in early pregnancy and risk of epilepsy in offspring. JAMA Neurol 2017;74:668-76.
5. Goode RH, Rettiganti M, Li J, et al. Developmental outcomes of preterm infants with neonatal hypoglycemia. Pediatrics 2016;138.
6. Kaiser JR, Bai S, Gibson N, et al. Association between transient newborn hypoglycemia and fourth-grade achievement test proficiency: a population-based study. JAMA Pediatr 2015;169:913-21.
7. Kerstjens JM, Bocca-Tjeertes IF, de Winter AF, Reijneveld SA, Bos AF. Neonatal morbidities and developmental delay in moderately preterm-born children. Pediatrics 2012;130:e265-72.
8. Tottman AC, Alsweiler JM, Bloomfield FH, Pan M, Harding JE. Relationship between measures of neonatal glycemia, neonatal illness, and 2-year outcomes in very preterm infants. J Pediatr 2017;188:115-21.
9. Oskoui M, Coutinho F, Dykeman J, Jette N, Pringsheim T. An update on the prevalence of cerebral palsy: a systematic review and meta-analysis. Dev Med Child Neurol 2013;55:509-19.
10. Fiest KM, Sauro KM, Wiebe S, et al. Prevalence and incidence of epilepsy: A systematic review and meta-analysis of international studies. Neurology 2017;88:296-303.
11. Maulik PK, Mascarenhas MN, Mathers CD, Dua T, Saxena S. Prevalence of intellectual disability: a meta-analysis of population-based studies. Res Dev Disabil 2011;32:419-36.
12. Bourne RRA, Flaxman SR, Braithwaite T, et al. Magnitude, temporal trends, and projections of the global prevalence of blindness and distance and near vision impairment: a systematic review and meta-analysis. Lancet Glob Health 2017;5:e888-e97.
